# Supplementary figures and images for: Transcriptomic analysis reveals high ITGB1 expression as a predictor for poor prognosis of pancreatic cancer
Source: PLoS One. 2022 Jun 1;17(6):e0268630. doi: 10.1371/journal.pone.0268630 (PMC9159604; doi:10.1371/journal.pone.0268630)

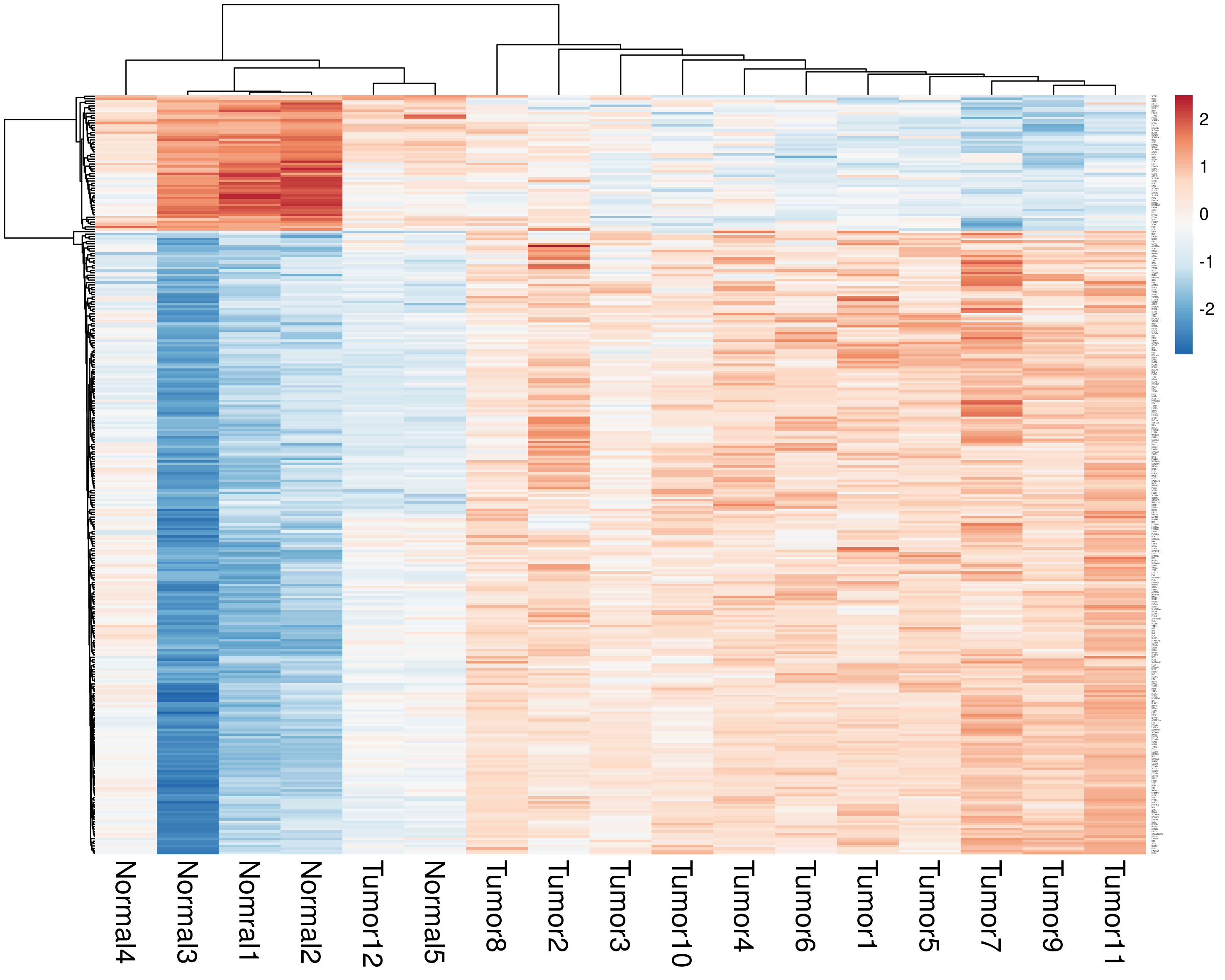

Supplement: S1 Fig — Genes with differential expression between the PDAC tissue and its adjacent pancreatic tissue were mapped and visualized on a heat map. (TIF) [file pone.0268630.s001.tif]
