# Supplementary material for: Transcriptomic analysis reveals high ITGB1 expression as a predictor for poor prognosis of pancreatic cancer
Source: PLoS One. 2022 Jun 1;17(6):e0268630. doi: 10.1371/journal.pone.0268630 (PMC9159604; doi:10.1371/journal.pone.0268630)
Supplement: Attachment — Submitted filename: Iwatate et al. 2021_Reviewers comments Dec 2021_2.docx [file pone.0268630.s003.docx]

PLOS one

**Transcriptomic analysis reveals high ITGB1 expression as a predictor for poor prognosis of pancreatic cancer.**

Using transcriptomic analysis Iwatate *et al.* have demonstrated that ITGB1 is highly expressed in a subset of patients with pancreatic cancer and this expression correlates with a poor prognosis. The authors used immunohistochemical analysis to support this finding.

The authors, state that ITGB1 is ‘an important gene for pancreatic cancer metastasis, progression and prognosis’ and that it ‘may be used as a drug target for pancreatic cancer’. These statements are somewhat supported by the data shown here, and is backed up by previously published work from other groups, but more *in vitro* and *in vivo* work would ultimately be needed to test these theories.

This work isn’t novel as it has previously been shown that ITGB1 is associated with poor survival previously (Sun *et al*. 2018), however, I appreciate that the work done here has been carried out on the under-sequenced Japanese population.

I have outlined some major and minor points below that would need to be addressed before this manuscript is accepted.

Major points

- Tables 1, 2 and 3 are missing from the manuscript and would need to be reviewed before acceptance.
- Concurrent with recent work by Sun *et al*. 2018 (Prognostic value of increased integrin-beta 1 expression in solid cancers; a meta-analysis). It would be good to show on the data set here the correlation with ITGB1 and OS (this may be shown in the missing figures), and discuss any differences.
- Figure 3. Images are of poor quality, with no scale bar making it difficult to interpret what is happening. The legend says that magnification was 160X – this doesn’t look to be correct. More description both on the figure and in the legend is required here.
- Figure 3. It had been mentioned that the tumour cell IHC scores were between 0-18. This data is not shown. Which sample set was this carried out on? The retrospective 107 patient samples or the 17 samples that were sequenced. Additionally, according to the methods, the maximum histoscore that could be achieved would be >80% (5) x staining higher than the control (3) = 15. However in the next the scores were between 0-18. This needs clarifying.

Minor points

- Ethics statement is missing from the author checklist (although it is present in the materials and methods.
- Sequencing data is not publically available, with no reason given.
- ‘Transcriptomic analysis of pancreatic cancer samples from the East Asian and Japanese population are lacking’. I appreciate that this is a novel data set that has been sequenced here, however, how does it compare to previous data sets? Are there specific genes/pathways differentially expressed here? Could this set be used for this?
- I’m not sure why the ‘traditional’ method of determining a histoscore wasn’t used here (intensity of 1-3 and % of tissue stained, giving a maximum histoscore of 300, compared to 15).
- Figure 2. Text mentions that ITGB1 was significantly correlated with the prognosis of pancreatic cancer and COL4A1 and COL4A2 were not. Data isn’t shown.
- Figure 4. The r values for the correlation between the RNA-seq and the IHC do not match up between figure and text (p=0.552 and p=0.542).
- Figure 4. It is unclear to what data the p values are relating. Additionally, 9.07 was mentioned on the graph, with no explanation in the figure legend. I’m assuming mean expression?
- Discussion – difference between the authors paper and that of Bailey *et.al;* the authors claim that the difference may be due to differences in the stromal content of the samples. Samples in the Bailey paper had a high epithelial content ≥40% (not 50% as stated in this manuscript). What is the stromal content of the samples used here?
- Overall the paper would benefit from being proof read

Typos

- RNA sequencing; the authors referred to a p value of 1/10000. The standard nomenclature would be p<0.0001
- IHC scoring of ITGB1 and related definitions; ‘Cases with an IHC expression score higher than the mean RNA expression level of ITGB1… Here it should be *ITGB1* due to references RNA expression.
- Patient backgrounds; IPMC is mentioned fir the first time, but hasn’t been defined.
- RNA sequencing; ‘Cytoscape was performed on these DEGs and the hub gene was found to be ITGB1…’ Here it should be *ITGB1*. And again in the last sentence of this section.
- Discussion; ‘The study for transcriptome inby Bailey et al.’ ‘inby’ as a typo and et al should be italicised.
