# Supplementary material for: Transcriptomic analysis reveals high ITGB1 expression as a predictor for poor prognosis of pancreatic cancer
Source: PLoS One. 2022 Jun 1;17(6):e0268630. doi: 10.1371/journal.pone.0268630 (PMC9159604; doi:10.1371/journal.pone.0268630)
Supplement: Attachment — Submitted filename: Iwatate et al. 2021_Reviewers comments Feb 2022.docx [file pone.0268630.s005.docx]

PLOS one

**Transcriptomic analysis reveals high ITGB1 expression as a predictor for poor prognosis of pancreatic cancer.**

The authors have addressed all my concerns, with the exception of the image quality of figure 3.

The authors propose that the image quality is ‘considered to be a problem in the journal review process’ that I should contact the publisher to confirm image quality.

I’ve not received a reply from the publisher regarding this figure, so cannot comment on the material within this image or conclusions derived from it.

Typo;

Page 24 in the discussion;

While it was reported that it functioned as a cell by ‘’construction of scaffolds’’ with integinand

Change in integrin and…

If the issue with image quality is resolved, I’d be happy to take another quick look at this section and recommend it for publication.
